# Arbuscular mycorrhizal fungi alleviates salt stress in *Xanthoceras sorbifolium* through improved osmotic tolerance, antioxidant activity, and photosynthesis

**DOI:** 10.3389/fmicb.2023.1138771

**Published:** 2023-03-16

**Authors:** Jianwei Zong, Zhilong Zhang, Peilu Huang, Yuhua Yang

**Affiliations:** ^1^College of Art, Henan University of Animal Husbandry and Economy, Zhengzhou, China; ^2^College of Forestry, Northwest A&F University, Yangling, China

**Keywords:** *Xanthoceras sorbifolium*, arbuscular mycorrhizal, photosynthesis, osmotic adjustment, antioxidant enzymes

## Abstract

Mycorrhizal inoculation was widely reported to alleviate the damage resulting from NaCl by various physiological ways. However, the symbiotic benefit under distant NaCl concentrations and the relationship among different responsive physiological processes were elusive. In this study, saline resistant plant *Xanthoceras sorbifolium* was selected as the experimental material and five concentrations of NaCl in the presence or absence of Arbuscular Mycorrhiza Fungi (AMF) were conducted, in order to understand the differences and similarities on the photosynthesis, antioxidant activity, and osmotic adjustment between arbuscular mycorrhizal (AM) plants and non-arbuscular mycorrhizal (NM) plants. Under low salt stress, *X. sorbifolium* can adapt to salinity by accumulating osmotic adjustment substances, such as soluble protein and proline, increasing superoxide dismutase (SOD), catalase (CAT) activity, and glutathione (GSH). However, under high concentrations of NaCl [240 and 320 mM (mmol·L^−1^)], the resistant ability of the plants significantly decreased, as evidenced by the significant downregulation of photosynthetic capacity and biomass compared with the control plants in both AM and NM groups. This demonstrates that the regulatory capacity of *X. sorbifolium* was limiting, and it played a crucial role mainly under the conditions of 0–160 mM NaCl. After inoculation of AMF, the concentration of Na^+^ in roots was apparently lower than that of NM plants, while Gs (Stomatal conductance) and Ci (Intercellular CO_2_ concentration) increased, leading to increases in Pn (Net photosynthetic rate) as well. Moreover, under high salt stress, proline, soluble protein, GSH, and reduced ascorbic acid (ASA) in AM plants are higher in comparison with NM plants, revealing that mycorrhizal symbiotic benefits are more crucial against severe salinity toxicity. Meanwhile, *X. sorbifolium* itself has relatively high tolerance to salinity, and AMF inoculation can significantly increase the resistant ability against NaCl, whose function was more important under high concentrations.

## 1. Introduction

Environmental stresses have many negative effects on the growth and development of many kinds of species. Salinity stress, which often occurs in arid and semi-arid regions and causes ionic, including osmotic and oxidant stresses (Rengasamy, 2006; Ashraf et al., 2008; Katerji et al., 2009; Munns, 2010), is one of the most complex and serious environmental problems. Over 6% of the world’s land area and approximately 20% of the farmland are currently influenced by saline soils (Munns, 2010). The effect of salinity, especially of high concentrations, can be firstly noticed at the whole plant level in the form of decline in biomass (Parida and Das, 2005). As for the concrete adverse influence of saline soil on plants, excessive Na^+^ could induce water deficiency by reducing the osmotic potential of soil, thus making it difficult for roots to absorb water (Rengasamy, 2006). Therefore, salt tolerance has universally been reported in relation to regulatory systems of ionic and osmotic homeostasis (Yeo, 1998; Zhu, 2002). Some biochemical molecules such as proline and proteins are vital properties for evaluating osmotic adjustment ability of plants (Zhu, 2002). Meanwhile, not only does salinity cause ionic and osmotic imbalance, but also alter various physiological and biochemical processes as evidenced by the parameters including enzyme activity, antioxidant substances, and photosynthetic rate (Iqbal et al., 2015; Elhindi et al., 2017). Excessive ROS under salinity is a result of disturbance of cellular homoeostasis, and it cause damages to protein oxidation, membrane integrity, and enzyme system, ultimately leading to the death of cells (Kohler et al., 2009; Ahmad et al., 2010; Alqarawi et al., 2014). Moreover, important antioxidant enzymes, especially peroxidase (POD), superoxide dismutase (SOD), and catalase (CAT), could rapidly clear deleterious $O_{2}^{\begin{array}{c} -\\ . \end{array}}$and promote the decomposition of H_2_O_2_, which was proved in the study of olive trees (Sofo et al., 2004). In terms of photosynthetic responses, except for the change in gas exchange characteristics, there are also distinct performances in light response curves under different NaCl treatments. The parameters such as apparent quantum efficiency (AQY), light saturation point (LSP), light compensation point (LCP), and maximum net photosynthetic rate (Pnmax) are significant in assessing the light energy utilization ability of plants under salt stress (Yu et al., 2019).

Arbuscular mycorrhizal fungi (AMF) have no strict host specificity, which can form beneficial symbiotic relationship with a variety of plants (Hooker et al., 1994). AMF has synergistic effect with many rhizosphere microorganisms, such as rhizobium and phosphor bacteria, and can alleviate many abiotic stress damage of plants (Xavier and Boyetchko, 2002). AMF colonization can facilitate the assimilation of mineral elements and enhance photosynthetic capability (Zhu et al., 2016), thus is beneficial for plant growth and biomass accumulation (Song et al., 2015; Mickan et al., 2016). In addition, AMF colonization also improves water use status in sweet basil under salinity (Elhindi et al., 2017). In recent years, there have been increasing studies on the effects of AMF on nutrition metabolism and stress resistance ability of plants (Latef and He, 2011; Rivero et al., 2015; Zhao et al., 2015). When exposed to salinity, positive effects of AMF inoculation were reported in many species, such as *Robinia pseudoacacia*, *Acacia gummifera*, *Retama monosperma*, and *Malus* × *domestica* Borkh (Yang et al., 2014; Chen J. et al., 2017; Fakhech et al., 2021), attributed to greater activity of antioxidant enzymes (Latef and He, 2011), accumulation of osmolytes (He et al., 2007), stimulation of phytohormone level (Abd Allah et al., 2015), and better acquisition of water and mineral nutrients.

*Xanthoceras sorbifolium* Bunge is an oil seed tree and is widely planted in the north of China (Fu Y. J. et al., 2008). As a native tree, *X. sorbifolium* performs well under abiotic stresses such as cold, drought, and salinity (Quanxin and Wenbin, 2014). Apart from the ecological functions and excellent resistant ability, *X. sorbifolium* also has valuable seeds, from which the oil extracted can be used to produce high-quality healthcare products, since the component in the oil is potentially effective for the treatment of Alzheimer’s disease, rheumatism, gout, and learning and memory impairment (Ji et al., 2017; Wu et al., 2018; Li et al., 2020). In our previous study, it was found that *X. sorbifolium* Bunge could cope with the salt stress by osmotic, enzyme, and photosynthesis regulations, which could effectively alleviate the damage caused by saline treatment (Zong et al., 2021). Wang et al. also investigated the physiological changes and molecular mechanisms underlying the responses of *X. sorbifolium* to salt and saline-alkali stress (Wang et al., 2020). The research about *X. sorbifolium* seedlings with AMF only answered the questions of mycorrhizal colonization and improvement in growth (Liu et al., 2020). To date, there have been few evidence confirming that symbiotic fungi will improve the condition of *X. sorbifolium* under salt stress, especially from the aspect of several physiological processes. In this study, the effect of AMF on the growth, physiology, and photosynthesis of *X. sorbifolium* under salt stress was studied. It may help develop thorough understanding of the mechanisms of symbiotic effect on promoting the application of AMF in saline soils.

## 2. Materials and methods

### 2.1. Plant material

The experiment was conducted at the research greenhouse with humidity of 60–75% and the temperatures was controlled at 18–35°C in Zhengzhou (113″ 80′ E, 34″ 80’ N), Northern China. Seeds of *Xanthoceras sorbifolium* Bunge were acquired from Jinshan Agricultural Science and Technology Co., Ltd. (Yangling, China). 10% (v/v) sodium hypochlorite aqueous solution (NaClO) was used for seed sterilization before the seeds were buried in sand in December 2019. In March 2020, healthy seedlings were selected and transplanted to flowerpots (inner diameter: 38.5 cm; height: 28.5 cm) containing 6.28 kg of substrate (The volume ratio of peat soil, coconut soil, and sand is 2:2:1). The pots were pre-sterilized by 10% NaClO. One seedling was planted in one pot. The substrate nutrient properties were measured (mg·g^−1^): organic matter, 51.2; ammonium nitrogen, 51.4; effect phosphorus, 37.0; and available potassium, 382.3. The substrate was autoclaved at 120°C for 2 h to remove all microorganisms. Then the plants were watered and weeded regularly in the greenhouse.

### 2.2. AMF inoculation and salinity inducement

In March 2020, for inoculation, prepared mycorrhizal inoculum was laid at the surrounding of roots. AMF (*Funneliformis mosseae*) was purchased from Root Mycorrhiza Research Institute of Changjiang University. Using white clover as a host, the microorganism agent was propagated by pot culture, and AMF inoculant was a mixture of mycorrhizal fungal spores, the root segment of the host plant, and the culture medium. The average spore density is 18–20 per 1 g of dried soil.

In August 2021, we set five NaCl stress concentrations in the experiment, including 0, 80, 160, 240, and 320 mM NaCl. Each treatment was divided in mycorrhizal treatment (AM) and non-mycorrhizal treatment (NM). A total of 10 treatment combinations were formed, and there were nine replicants for each treatment, with a total of 90 pots. In the experiment, the leaves of the third to fifth segments at new shoots were selected as samples, and at least three biological repeats were used for each determination.

The stress treatment was induced by NaCl solution. In order to avoid the salt shock, the solution was gradually added to the target concentration (irrigated four times, 100, 200, 300, and 400 mL, respectively). At the same time, a tray was put under the pot to collect the outflow solution, and the solution was poured back into the flowerpot in time after each watering. After adding salt, the NaCl content of the substrate was re-determined, and the subsequent experiment was continued after all treatments reach the set concentration. After 8 days of salt treatment, the experiments below were conducted in order.

### 2.3. Assessment of root colonization infection

Fresh roots were used to determine mycorrhizal colonization. Distilled water was used in washing process, and the roots were cut into about 1 cm length pieces. Following the method in Phillips (1970), the root pieces were more transparent after soaking in 5% potassium hydroxide (KOH), then they were acidified in 1% hydrochloric acid solution (HCl) and stained in 0.05% trypan blue dissolved in lactophenol before observation under an optical microscope. The gridline intersect method (Giovannetti and Mosse, 2010) was applied to determine the mycorrhizal colonization.

### 2.4. Determination of biomass

Deionized water was used to carefully clean the leaves, shoot, and the root of the *X. sorbifolium*. Three plants were randomly selected with consistent great growth in every treatment. After leaves, roots, and shoots were thoroughly dried at 80°C in a heating oven for about 48 h, dry weight was noted.

### 2.5. Concentration of Na^+^ and K^+^

The harvested fresh roots were dried and grounded into powder. Each sample (0.1 g) was digested with a mixture of 8 ml nitric acid (HNO_3_) and 2 ml perchloric acid (HClO_4_), and the solution was heated to 220°C. After cooling to 27°C, the extraction solution was diluted. The concentrations of Na^+^ and K^+^ were determined by using Z-2000 atomic absorption spectrophotometer (Shimadzu, Japan).

### 2.6. Determination of relative electrical conductivity and malondialdehyde

Relative electrical conductivity (REC) was determined in fresh by employing the methods of Maribel (Dionisio-Sese and Tobita, 1998). 0.2 g fresh leaf samples were cut into 6 mm length segments and soaked in deionized water. The test tubes containing samples stood still for 12 h before a conductivity meter DDS-307 (Leici Corporation, China) was employed to measure the first conductivity value (S_1_). The homogenate was heated in boiling water for 20 min to destroy the tissue cells, then cooled down to 27°C and ultimately all electrolytes were released. Afterward, the second conductivity value (S_2_) was recorded. The REC was expressed following the formula:


(1) $$Relativeelectricalconductivity=\frac{S1}{S2}\times 100\%$$

To determine the concentration of malondialdehyde (MDA), a method described by Stewart and Bewley was applied (Stewart and Bewley, 1980). In short, 0.5 g of each fresh leaf sample was grounded in 10 mL of 10% trichloroacetic acid solution. After centrifugation at 14,000 × *g* for 7 min, the supernatant from crude extract was collected and thoroughly mixed with 0.6% of thiobarbituric acid and heated up to 95°C for 30 min, and then the reaction was suspended by an ice bath. The mixture was then centrifuged at 10,000 × *g* for 10 min, and the absorbance of the supernatant was recorded at 532 and 660 nm.

### 2.7. Determination of soluble proteins and proline

Soluble protein contents were determined by using the method of Bradford (1976). Bovine serum albumin was applied as the determination standard. Fresh leaf samples (0.5 g) from each treatment were collected, and grinded in 2 mL of distilled water. Afterward, the mortar was rinsed using 6 mL of distilled water. The homogenate and washing liquid were poured into the tube and then centrifuged at 4,000 × *g* for 10 min. The resulting supernatant was moved into a 10 mL graduated bottle, which was then filled to its scale and then swayed evenly. Moreover, the extract of 0.1 mL was transferred into a tube with stopper. 5 ml of Coomassie bright blue G-250 was added into the tube and mixed thoroughly. Standing still for 2 min, the absorbance of final solution was measured at 595 nm by using a spectrophotometer (UV-2100, UNIC, United States).

Proline concentration was measured by using method illuminated by Bates (Bates et al., 1973). 0.3 g of leaves was grounded in 5 mL of sulfosalicylic acid solution (3%) and then the homogenate was centrifugated at 10,000 × *g* for 15 min. 2 ml glacial acetic acid and 3 ml acid ninhydrin were added in to 2 ml of the supernatant and the mixture was heated at 100°C for 1 h. Thereafter, the tube was placed into an ice bath to finish the reaction. Proline was extracted with 4 ml of toluene. The absorbance was measured at 520 nm with toluene as the standard.

### 2.8. Determination of glutathione and reduced ascorbic acid

To determine glutathione (GSH) and ascorbic acid (ASA), the standard curves of ASA and GSH concentrations were estimated respectively, and then ASA and GSH in 0.5 g fresh leaf samples were extracted using 5% metaphosphoric acid and 1 mM ethylene diaminetetraaceticacid (EDTA) at 11,500 × *g* centrifugation for 15 min. Then, after collecting the supernatant, ASA and GSH were determined by the method depicted by foyer (Foyer et al., 1983) and Yu (Yu et al., 2003). The content of ASA was estimated employing spectrophotometry method. The content of GSH was calculated by subtracting GSSG (oxidized glutathione) from the total GSH.

### 2.9. Gas exchange parameters

Taking Li-6800 Portable Photosynthesis System (LI-COR, Lincoln, Nebraska) as a measuring tool, net photosynthetic rate (Pn), transpiration rate (E), stomatal conductance (Gs), and intercellular CO_2_ concentration (Ci) were evaluated in a sunny day. The CO_2_ concentration of the reference chamber was set as 400 μmol·mol^−1^, the temperature of the leaf chamber was set at 30°C, the relative humidity of the leaf chamber was set as 50%, and the light intensity was set as 1,200 μmol·m^−2^ s^−1^ photosynthetically active radiation (PAR). The fully developed leaves at the same leaf position from each treatment were used for gas exchange parameter measurements.

### 2.10. Pn-PAR curves and photosynthetic parameters

Using LI-6800 Portable Photosynthesis System analyzer to measure the Pn-PAR curve, PAR was set as: 1,800, 1,500, 1,200, 900, 600, 300, 200, 150, 100, 70, 30, and 0 μmol·m^−2^ s^−1^. Before each measurement, the measured leaves were induced for about 20–30 min at light intensity of 1,200 μmol·m^−2^ s^−1^. CO_2_ concentration was controlled by an external CO_2_ small cylinder, and the concentration was set to 400 μmol·mol^−1^, which was similar to the atmospheric CO_2_ concentration. The light compensation point (LCP, μmol·m^−2^ s^−1^), light saturation point (LSP, μmol·m^−2^ s^−1^), apparent quantum efficiency (AQY, μmol·m^−2^ s^−1^), and maximum net photosynthetic rate (Pnmax, μmol·m^−2^ s^−1^) dark respiration rate (Rd., μmol·m^−2^ s^−1^) were obtained according to the modified rectangular hyperbola model since it has a larger *R*^2^ value compared to the other three models (Li et al., 2019).


(2) $$Pn\left(I\right)=\alpha \frac{\left(1-\beta I\right)}{\left(1+\gamma I\right)}I-Rd$$

### 2.11. Determination of antioxidant enzymes.

For enzyme assays, the leaves of *X. sorbifolium* were picked and cleaned in deionized water. The fresh leaf samples (0.5 g) were first stored in a freezer and then grounded in 5 ml of icy 50 mM potassium phosphate buffer (pH = 7.0) consisting of 1 mM EDTA, 1% (w/v) polyvinylpolypyrrolidone (PVPP) and 2 mM ascorbic acid (ASA) into homogenate using prechilled mortars and pestles. The mixture was centrifuged at 12,000 × *g* for 20 min and the resulting supernatant was collected for determining the activities of SOD, POD, and CAT.

Superoxide dismutase activity was assayed based on the ability to inhibit the reduction of nitro blue tetrazolium (NBT) on the basis of the method expounded by Rossum (van Rossum et al., 1997). To a buffer (2.8 mL) including 50 mM potassium phosphate buffer (pH 7.8), 13 mM methionine, 75 mM NBT, 0.1 mM EDTA, and 0 or 100 μl of the enzyme extract solution was added 20 μM riboflavin. After adding riboflavin, the reaction started. The tubes were shaken and illuminated in a light box with two 15 W fluorescent lamps for 20 min. This reaction was permitted to run for 10 min, afterward the light was switched off and the reaction stopped. The absorbance was read at 560 nm. One unit of SOD activity was defined as the quantity of enzyme required to cause 50% inhibition in comparison with tubes lacking enzymes.

Catalase activity was estimated as the consumption of H_2_O_2_ by the method illustrated by Cohen and Somerson (1969). The reaction buffer (3 mL) consists of 50 mM phosphate buffer (pH = 7.0), 200 mM hydrogen peroxide (H_2_O_2_), and 100 μl enzyme extracts solution. H_2_O_2_ decrease was recorded as the decline in optical density at 240 nm for 1 min. One unit of CAT activity was calculated as the decrease of absorbance at 240 nm by 0.1 unit per min.

Peroxidase activity was measured according to the guaiacol oxidation method described by Lundquist and Josefsson (1971), with some modifications. The assay mixture (3 mL) contained 10 mM potassium phosphate buffer (pH = 7.0), 0.33 mM guaiacol, and 100 μL of enzyme extract solution. The reaction started with 40 mM H_2_O_2_. The oxidation of guaiacol in the presence of H_2_O_2_ was estimated at 470 nm over a 2 min interval. One unit of peroxidase activity was defined as the quantity of 1% (w/v) guaiacol oxidized per minute.

### 2.12. Statistics

The data were analyzed by the SPSS 25.0 statistical software (SPSS Inc., Chicago, IL, United States). According to one-way ANOVA and post comparisons (Duncan’s test, *p* < 0.05), the significance was calculated and labeled if compared to controls. Figures were drawn in Origin 2021 software (ORIGIN, Massachusetts, United States). Principal component analysis (PCA) was performed to characterize the salt tolerance of the *X. sorbifolium*.

## 3. Results

### 3.1. Root AM colonization

Non-inoculated plants did not show any colonization, root colonization by the arbuscular mycorrhizal fungi was observed with highest without salt stress. Increased salinity significantly reduced the colonization rate of arbuscular, hyphae, and colonization rate compared to control plants in AM groups (*p* < 0.05), while the vesicle colonization showed an increasing trend (Table 1).

**Table 1:** Mycorrhizal fungal hyphae, vesicles, and arbuscules colonization of the experimental plants.

NaCl (mM)	Arbuscular (%)	Vesicles (%)	Hyphae (%)	Colonization rate (%)
0	45.33 ± 4.04a	27.00 ± 2.65b	66.00 ± 4.36a	46.11 ± 1.9a
80	38.00 ± 2.65b	28.33 ± 4.04b	54.00 ± 3.61b	40.11 ± 1.84b
160	32.67 ± 2.52bc	30.67 ± 4.04ab	46.33 ± 3.21c	36.56 ± 2.83c
240	28.33 ± 2.52c	33.67 ± 2.08ab	39.00 ± 2.00d	33.67 ± 2.03d
320	22.00 ± 3.61d	34.33 ± 3.06a	33.00 ± 2.65e	29.78 ± 3.10e

Values marked with different letters are significantly different at *p* < 0.05.

### 3.2. Biomass

**Figure 1:**
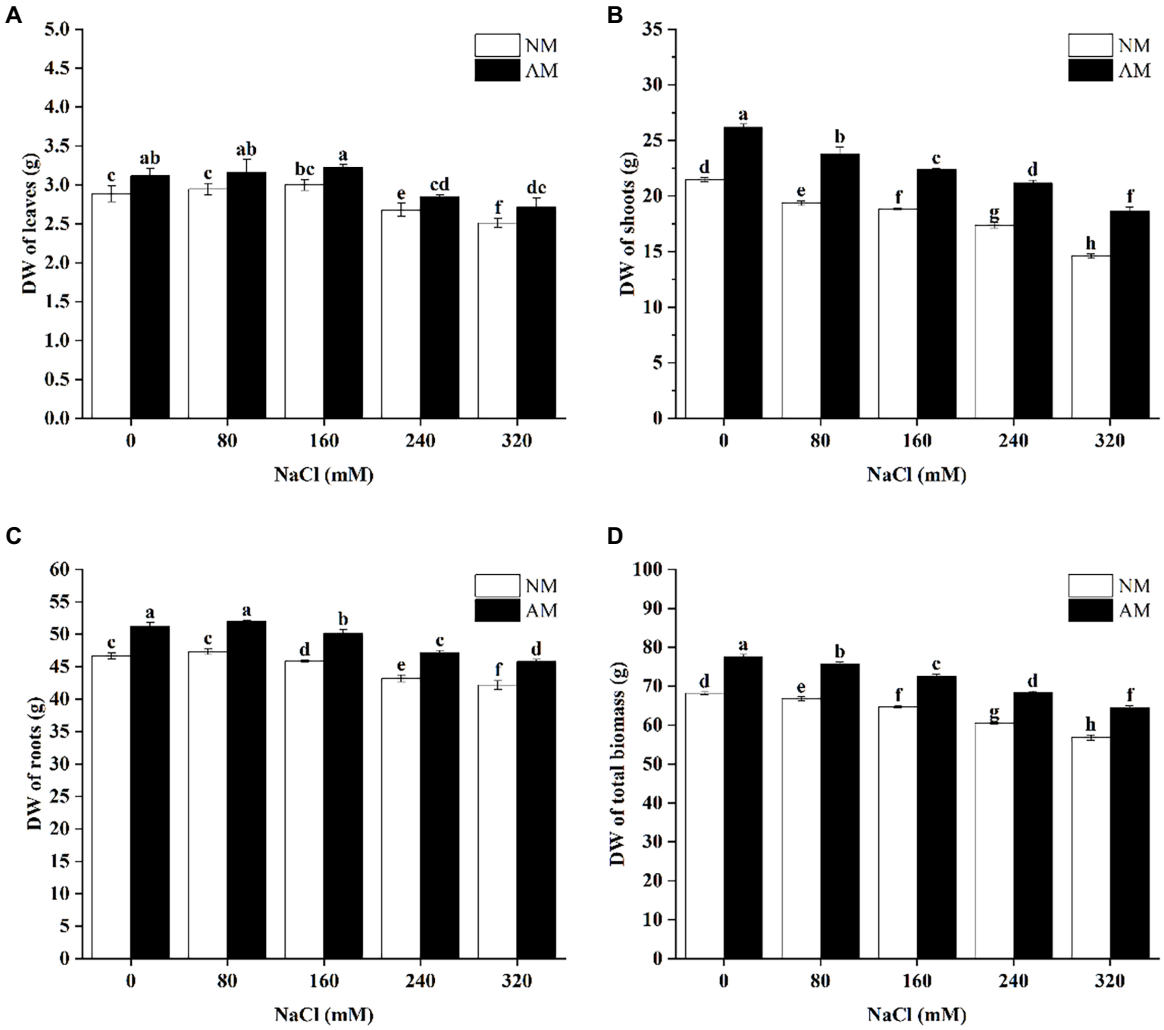
The dry weights (DWs) of the leaves **(A)**, shoots **(B)**, roots **(C)**, and the total biomass **(D)** in *Xanthoceras sorbifolium* were affected by NaCl stress levels and AMF inoculation. Different letters indicate significant differences (*p* < 0.05). AM, arbuscular mycorrhizal; NM, non-mycorrhizal.

The DWs of the leaves, shoots, roots, and total biomass of AM plants were significantly higher than those of NM plants at corresponding salinity levels (Figure 1). Nevertheless, compared with corresponding control plants, the DWs of roots and leaves in AM and NM group did not decrease significantly at 80 mM NaCl (*p* > 0.05). However, when exposed to NaCl concentrations above 160 mM, the DWs of both leaves and roots declined significantly (*p* < 0.05), but other than this, compared with NM treatment, the DWs of the leaves, shoots, roots, and total biomass of AM plants significantly increased by 8.23, 27.63, 8.63, and 13.51% at 320 mM NaCl, respectively.

### 3.3. Concentration of Na^+^ and K^+^

**Figure 2:**
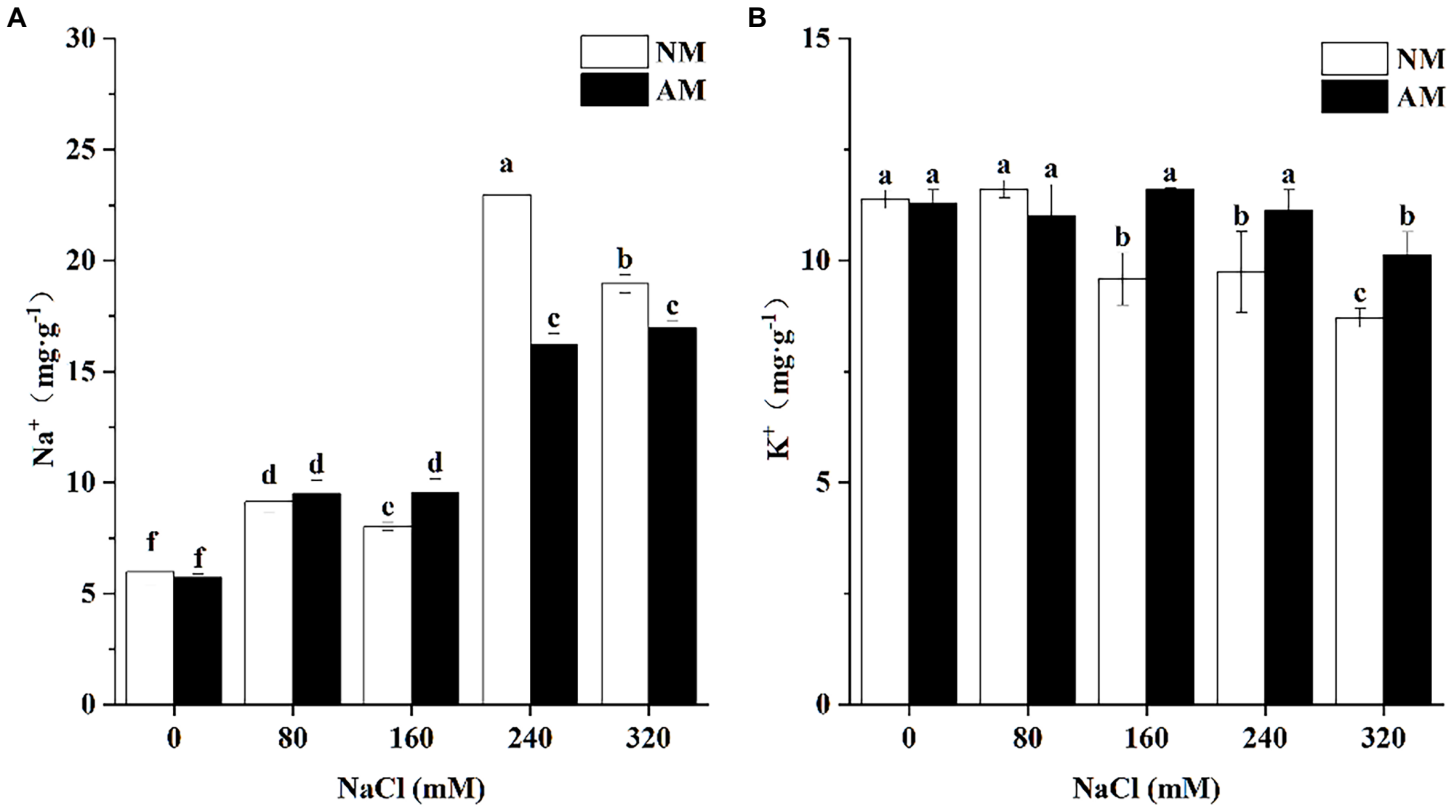
Concentration of Na^+^**(A)** and K^+^ **(B)** in the roots of *Xanthoceras sorbifolium*. Plants were affected by salt stress levels and AMF inoculation. AM, arbuscular mycorrhizal; NM, non-mycorrhizal. Different lowercase letters indicate significant differences among different NaCl concentration (Duncan’s test, *p* < 0.05).

Salinity can result in the accumulation of Na^+^ in both NM plants and AM plants (Figure 2A). No significant differences of Na^+^ concentration in NM plants and AM plants at 0 and 80 mM NaCl were observed. However, when treated with 240 and 320 mM NaCl, the Na^+^ concentration in AM plants was significantly lower than that of NM plants (*p* < 0.05), by 41.61 and 11.82%, respectively, (Figure 2A). It is worth noting that K^+^ concentration was less than that of NM plants at 0 and 80 mM NaCl, but under higher concentrations (160, 240, and 320 mM NaCl), the value was greatly improved by AM symbiosis (Figure 2B).

### 3.4. Relative conductivity and malondialdehyde

**Figure 3:**
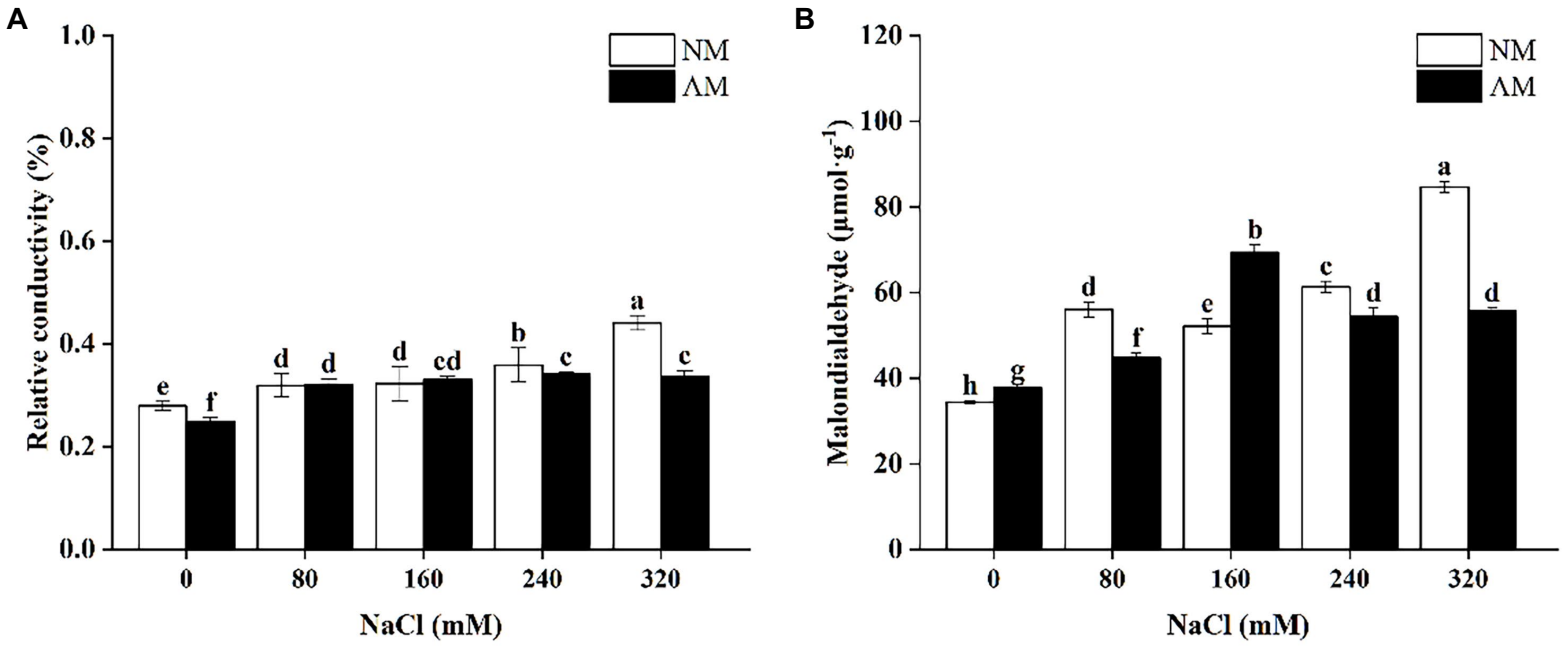
Relative conductivity **(A)** and Malondialdehyde **(B)** in *Xanthoceras sorbifolium* were affected by salt stress levels and AMF inoculation. AM, arbuscular mycorrhizal; NM, non-mycorrhizal. Different lowercase letters indicate significant differences among different NaCl concentration (Duncan’s test, *p* < 0.05).

Figure 3A shows that upon exposed to 0, 240, and 320 mM NaCl, there is a significant decline in the REC of AM plants, which were 10.74, 4.83, and 23.66% lower than NM plants, respectively (*p* < 0.05). MDA of AM plants increased under low salt stress and reduced when NaCl was supplied more than 160 mM. Besides, MDA of NM plants increased continuously except for 160 mM NaCl (Figure 3B). In addition, under 80, 240, and 320 mM NaCl, MDA of AM plants under salt stress were significantly lower than those of NM plants (*p* < 0.05). Surprisingly, mycorrhizal colonization caused the largest reduction by 34.08% at 320 mM NaCl in comparison to NM plants.

### 3.5. Soluble protein and proline

**Figure 4:**
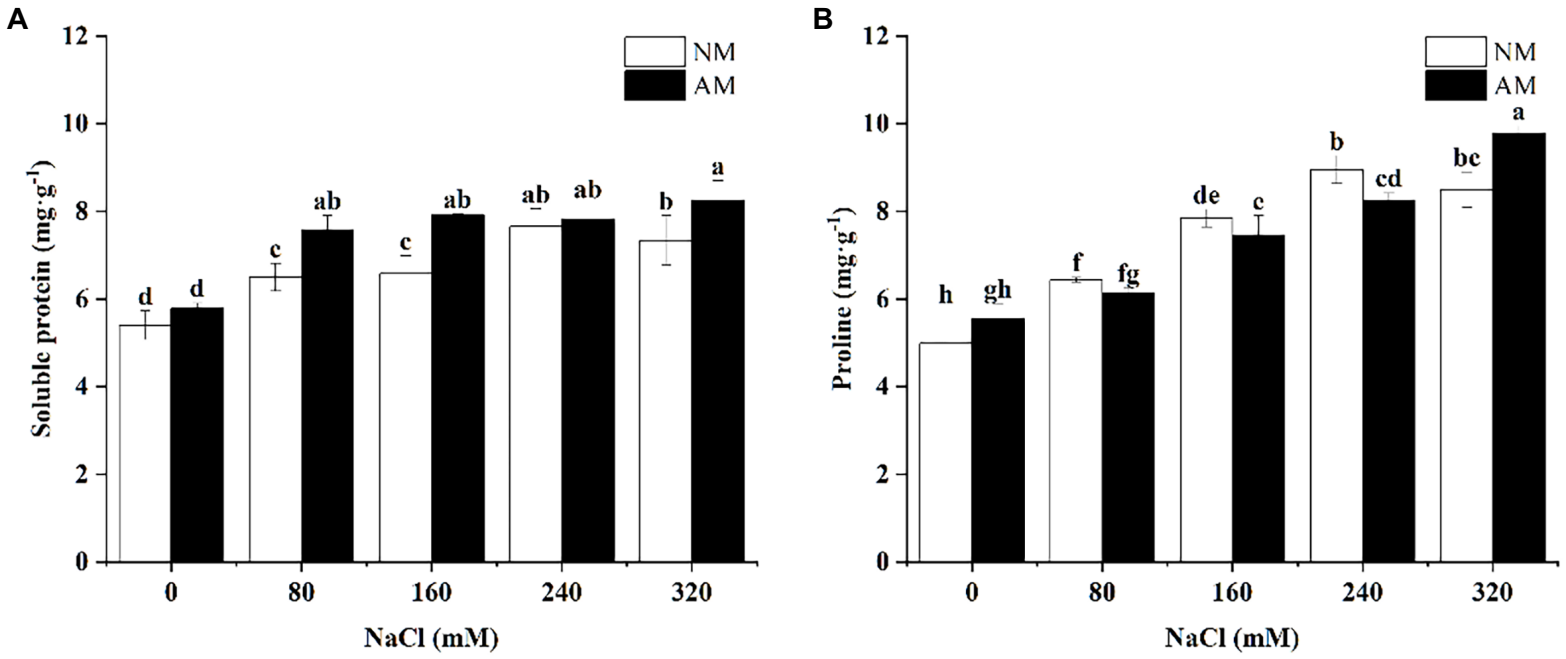
Soluble protein **(A)** and Proline **(B)** in *Xanthoceras sorbifolium* were affected by salt stress levels and AMF inoculation. AM, arbuscular mycorrhizal; NM, non-mycorrhizal. Different lowercase letters indicate significant differences among different NaCl concentration (Duncan’s test, *p* < 0.05).

Increasing salinity caused significant increase in soluble protein of AM and NM plants versus corresponding control plants (*p* < 0.05; Figure 4). Besides, inoculation of AMF can improve the amounts of soluble protein and proline of *X. sorbifolium* upon exposure to salinity. As shown in Figure 4A, the soluble protein of NM plants reached to the maximum at 240 mM NaCl, but that of AM plants peaked at 320 mM NaCl. The peak of proline in both AM and NM plants was close to that of soluble protein, but other than this, under 320 mM NaCl, proline in AM plants significantly increased by 15.10% in comparison with NM plants (*p* < 0.05). Interestingly, compared with NM, proline content of AM plants was lower under 80, 160, and 240 mM.

### 3.6. Glutathione and reduced ascorbic acid

**Figure 5:**
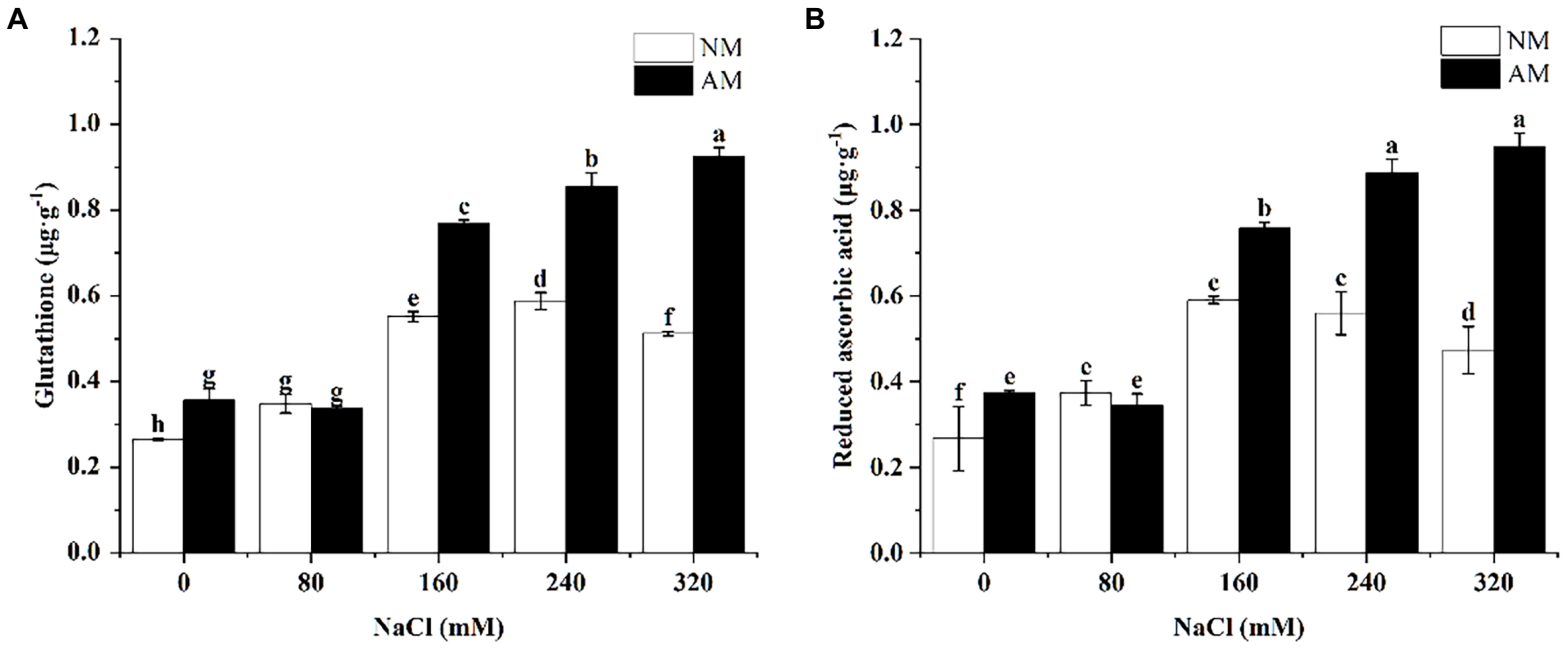
Glutathione **(A)** and reduced ascorbic acid **(B)** in *Xanthoceras sorbifolium* were affected by salt stress levels and AMF inoculation. AM, arbuscular mycorrhizal; NM, non-mycorrhizal. Different lowercase letters indicate significant differences among different NaCl concentration (Duncan’s test, *p* < 0.05).

Figure 5A indicates that except for 80 mM, arbuscular mycorrhizal yielded a significant increase in the GSH level in comparison to NM plants (*p* < 0.05). What is more, colonization with AMF caused dramatic increase in the GSH content under higher NaCl concentrations (160, 240, and 320 mM). A similar trend for ASA was observed (Figure 5B). The maximum of NM plants occurred at 160 mM, but after inoculation, the peak value appeared under 320 mM NaCl treatment.

### 3.7. Photosynthesis

**Figure 6:**
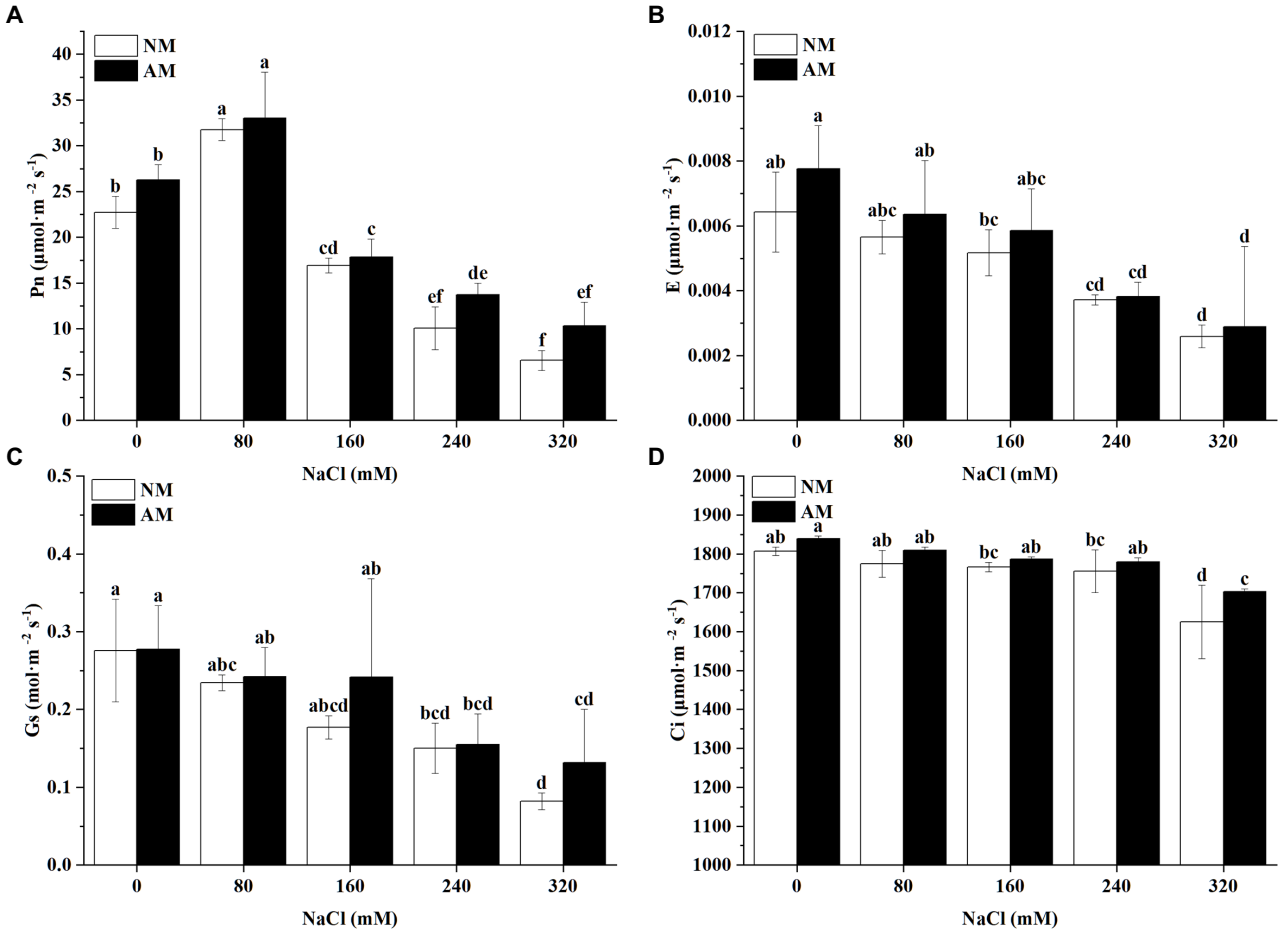
**(A)** Pn, **(B)** E, **(C)** Gs, and **(D)** Ci in *Xanthoceras sorbifolium* were affected by salt stress levels and AMF inoculation. AM, arbuscular mycorrhizal; NM, non-mycorrhizal. Different lowercase letters indicate significant differences in concentration (Duncan’s test, *p*  < 0.05).

Salinity negatively affected the Gs and E in NM and AM plants (Figure 6). Nevertheless, Pn showed an upward trend at 80 mM NaCl, followed by a return to minimum levels at the highest concentration. Pn of NM plants reached its maximum value at 80 mM NaCl, 39.74% higher than the control plants in NM group (Figure 6A). Notably, mycorrhizal colonization markedly improved Gs, E and Pn in comparison to the non-mycorrhizal plants. The mycorrhization process led to a strong increase of Gs in comparison with NM plants when the salinity was supplied at 320 mM (Figures 6A–C). Whether inoculated or not, increasing salt stress caused the decrease in Ci (Figure 6D). Indeed, the mycorrhization in itself induced a notable increase in Ci, especially under high concentrations.

### 3.8. Light response

**Figure 7:**
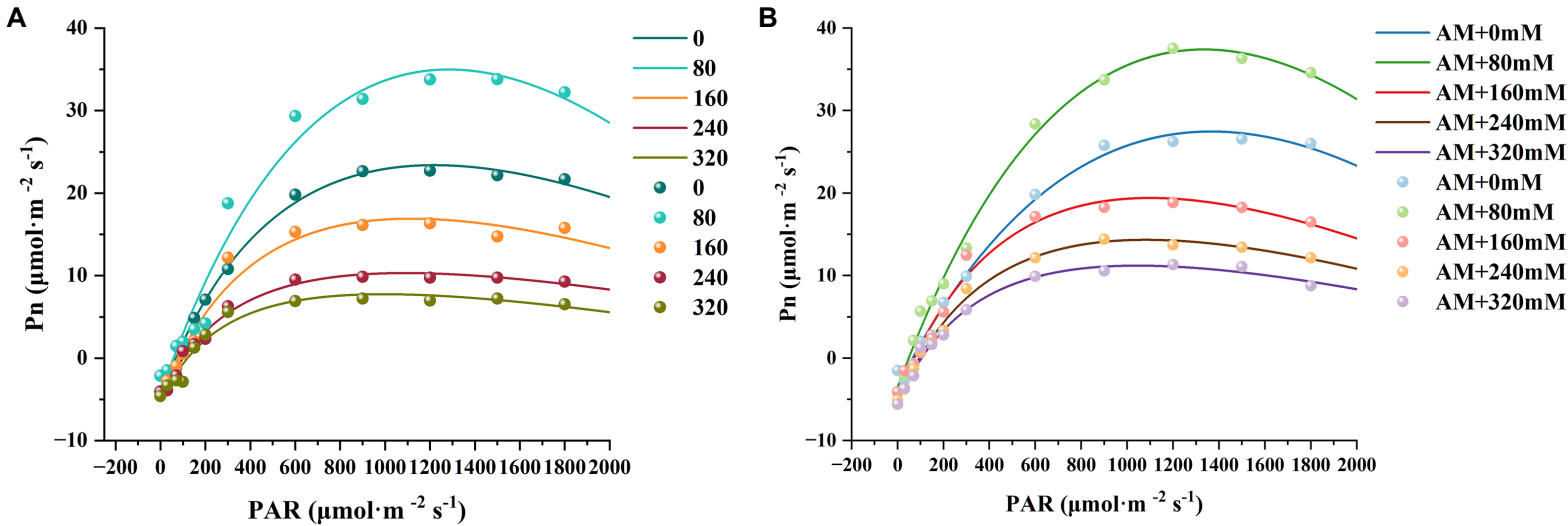
Light response curves of *Xanthoceras sorbifolium* treated with different salt concentrations. NM **(A)**, non-mycorrhizal; AM **(B)**, arbuscular mycorrhizal.

As shown in Figure 7, Pn initially increased sharply and then reached a plateau upon increasing PAR. No matter low and high NaCl treatment, photosynthetic ability of *X. sorbifolium* can be enhanced by AM inoculation. Surprisingly, when PAR was set during 800–1,800 μmol·m^−2^ s^−1^ (close to LSP), Pn of AM plants obviously exceeded NM plants, and the difference was most prominent at 80 mM NaCl. It revealed that the effect of inoculation was more vital to photosynthesis under 80 mM NaCl. More likely, photosynthetic performance is sensitively affected by AMF.

From Table 2, it can be seen that AQY, Pnmax, and LSP of AM and NM plants all showed an increasing trend under low salt stress and then decreased under high salt stress. AQY of AM plants was greater than NM plants at 0 and 80 mM NaCl. However, when treated with higher concentrations, AM plants failed to change AQY compared with NM plants, which showed that the promotion of AMF on AQY was not obvious under severe salt stress. It was worth noting that the Pnmax, and LSP of AM plants were higher than that of NM plants at either treatment, and the values of AM plants increased by 44.33 and 6.60% even under 320 mM NaCl. Among all salt stress treatments, the value of LCP in AM plants was lower than that of NM plants, suggesting that AM inoculation can increase the minimum light energy radiation available to the leaves.

**Table 2:** The light compensation point (LCP), light saturation point (LSP), apparent quantum efficiency (AQY), and maximum net photosynthetic rate (Pnmax) of *Xanthoceras sorbifolium* were affected by salt stress levels and AMF inoculation.

AMF inoculation	NaCl (mM)	AQY μmol ·mol^−1^	Pnmax μmol·m^−2^ s^−1^	LSP μmol·m^−2^ s^−1^	LCP μmol·m^−2^ s^−1^
NM	0	0.074 ± 0.005abc	23.378 ± 0.51bc	1221.918 ± 30.62bc	68.261 ± 2.13def
	80	0.077 ± 0.003ab	34.648 ± 0.989a	1271.287 ± 47.214ab	55.927 ± 4.258 fg
	160	0.071 ± 0.002abc	16.904 ± 0.811d	1117.605 ± 40.357 cd	81.560 ± 12.055 cd
	240	0.064 ± 0.002bc	10.381 ± 2.378ef	1084.547 ± 36.298 cd	104.031 ± 7.613b
	320	0.060 ± 0.002c	7.772 ± 0.933f	1024.945 ± 117.382d	120.088 ± 1.874a
AM	0	0.077 ± 0.019ab	27.713 ± 0.568b	1269.429 ± 17.329ab	63.096 ± 5.043efg
	80	0.083 ± 0.005a	37.496 ± 8.044a	1398.624 ± 141.552a	50.062 ± 2.992 g
	160	0.071 ± 0.006abc	19.498 ± 0.486 cd	1119.946 ± 87.368 cd	72.265 ± 4.492de
	240	0.064 ± 0.013bc	14.478 ± 2.061de	1096.521 ± 82.043 cd	93.935 ± 16.162bc
	320	0.061 ± 0.005bc	11.217 ± 2.388ef	1092.542 ± 101.154 cd	106.733 ± 6.196b

AM, arbuscular mycorrhizal; NM, non-mycorrhizal. Different lowercase letters indicate significant differences in concentration (Duncan’s test, *p* < 0.05).

### 3.9. Antioxidant enzymes

**Figure 8:**
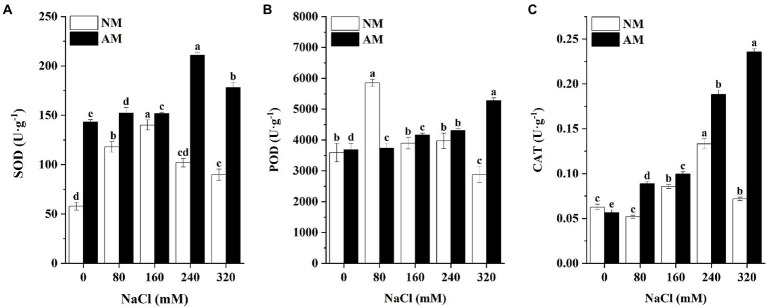
The SOD activity **(A)**, POD activity **(B)**, and CAT activity **(C)** in *Xanthoceras sorbifolium* leaves were affected by salt stress levels and AMF inoculation. AM, arbuscular mycorrhizal; NM, non-mycorrhizal. Different lowercase letters indicate significant differences in concentration (Duncan’s test, *p* < 0.05).

Salinity and AMF symbiosis significantly altered the antioxidant enzyme activities (Figure 8). The mycorrhization process led to a strong increase in SOD activity. Unlike, SOD showed an upward trend at low-to-medium salt concentrations up to 160 mM NaCl, followed by a return to minimum levels at the highest concentration. Moreover, POD activity of NM plants showed the highest value under salt stress of 80 mM and minimum value at 320 mM NaCl. By contrast, the POD increased gradually in salt affected AM plants. Generally, POD of AM plants under most of concentrations is higher than NM plants. In terms of CAT activity, it increased sharply after inoculation, especially under high salt concentrations (320 mM). Salinity negatively affected CAT activity, causing reductions in the CAT of AM plants of 56.76, 75.68, 232.43, and 316.22% at 80, 160, 240, and 320 mM NaCl versus control plants in AM treatment, respectively. Especially, CAT activity of AM plants was very high compared with non-mycorrhized ones at the corresponding salinity level. On the other hand, the increase in CAT activity resulted from AMF symbiosis at the level of 320 mM NaCl was at least 3-times more versus non-mycorrhized ones (Figure 8).

### 3.10. Principal component analysis

**Figure 9:**
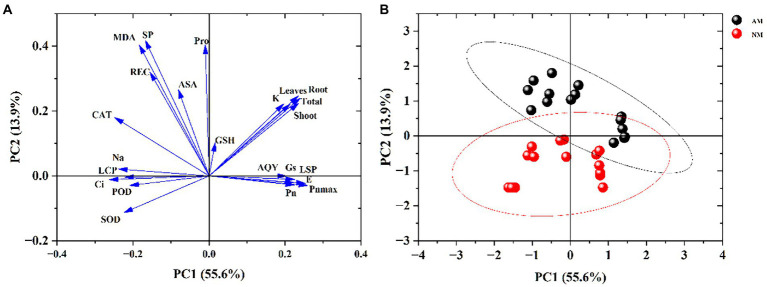
Principal components analysis of the whole data involving growth, physiology, photosynthesis, antioxidative enzyme activities and inorganic ion in *Xanthoceras sorbifolium* after salt stresses. Loading plot **(A)**; Score plot **(B)**.

In this study, 23 growth, photosynthesis, antioxidant enzyme activity, inorganic ions, and physiological parameters were analyzed with principal component analysis (PCA). Figure 9A shows that photosynthetic parameters and biomass are the main drivers of the first component (PC1). In contrast, proline, soluble protein and MDA are the main drivers of the second component (PC2). Figure 9B shows the score plot of PC1 and PC2 separating NM and AM plants under salt stress. The results indicated that the plants inoculated with mycorrhizal fungi and the plants without mycorrhizal fungi could be well divided into two groups, which was mainly determined by the significant changes in photosynthesis and biomass of AM and NM plants. The changes in proline, soluble protein, and other indicators also participated in and affected the distribution of AM and NM plants on the score plot. Finally, this result revealed that AMF inoculation has a remarkable impact on the resistance of *X. sorbifolium* to salt stress.

## 4. Discussion

Many possible physiological and biochemical processes about the interaction of AMF and salinity have been reported to test the effect of certain kind of mycorrhizal and the concrete modulate mechanism (Türkmen et al., 2008; Zai et al., 2014; Gao et al., 2020). Moreover, mycorrhizal inoculation can reduce Na^+^ uptake, change the osmotic equilibrium of roots, and ultimately improve the salt tolerance of plants (Garg and Manchanda, 2010). In the meanwhile, AMF can retain Na^+^ in the hyphae outside the root and reduce the amount of Na^+^ entering the plant root (Wu et al., 2015). Our results revealed that the difference between AMF-inoculated seedlings and non-inoculated ones can be partly explained by the symbiotic benefit, which can be evaluated from membrane permeability and the photosynthetic, osmotic, and antioxidant systems. Under the harsh environment of saline soils, mycorrhizal fungi may be an accessible strategy to alleviate the damage of salt stress and improve the growth condition in *X. sorbifolium.*

Saltiness diminishes colonization by smothering hyphal development, sporulation, and spore germination (Juniper and Abbott, 2006). In the experiments reported here, salinity caused a reduction in mycorrhizal colonization in *X. sorbifolium*, indicating that salinity inhibited the spread of mycorrhizal colonization (Table 1). In fact, it can be seen that the colonization rate was significantly lower as a result of high salt stress (240 and 320 mM), as compared with control plants in AM treatment (Table 1). Similar results have been seen on lettuce and *Trigonella foenum-graecum* (Aroca et al., 2013; Evelin and Kapoor, 2014). In addition, the change in plant biomass is the most obvious appearance reflecting plant tolerance under abiotic stress and the symbiotic benefits of AMF (Heikham et al., 2009; Ruiz-Lozano et al., 2012; Porcel et al., 2016). In our study, although the DWs of leaves, shoots, root, and total biomass in both NM and AM plants decreased when exposed to increasing salt pressure, above parameters were significantly enhanced by mycorrhizal across all salinity levels, suggesting that the mycorrhizal can alleviate the damage from salt stress in *X. sorbifolium* to a certain extent. Beneficial impact of AM on biomass creation in plants when salt pressure is present has also been reported in other plant species, including tomato, rice, lettuce, maize, black locust, and poplar (Hajiboland et al., 2010; Min et al., 2011; Aroca et al., 2013; Wu et al., 2015; Porcel et al., 2016; Chen J. et al., 2017). AMF did not change the downward law that the biomass of *X. sorbifolium* upon exposure to salinity, while among various biomass indicators, the increase of biomass of shoot in AM plants is most pronounced at high concentrations. The promotion (symbiotic benefit) of leaves, shoot, root and total biomass resulted from AMF inoculation was stable under different NaCl concentrations, and AM colonization decreased gradually, which showed that the colonization is not a decisive factor to symbiotic benefit. Further research on the decisive factor of AMF symbiotic benefit needs to be established.

Absorption and distribution of Na^+^ is the first step of salinity influence, and it can reflect the salt tolerance of plants to a certain degree (Porcel et al., 2016). Previous studies have reported a decrease in Na^+^ accumulation in AM plants especially in roots (Chen J. et al., 2017). As expected, cytosolic Na^+^ concentration in the roots of AM plants is significantly lower than that of NM plants upon exposure to salinity, but this situation only occurs in the NaCl concentrations of 240 and 320 mM, suggesting that mycorrhizal inoculation can inhibit the accumulation of Na^+^ into the root especially under high concentrations (Parihar et al., 2020). In contrast, compared with NM plants, K^+^ concentration of AM plants significantly increased under 160, 240, and 320 mM. Several researchers reported that K^+^ concentration increased whereas Na^+^ concentration decreased after inoculation (Al-Karaki et al., 2001; Garg and Manchanda, 2010; Wang et al., 2022), and our study observed a similar inclination. The mycorrhizal mainly alleviates the ionic toxicity by decreasing Na^+^ uptake and promoting accumulation of K^+^ in the root of *X. sorbifolium.*

Salt stress will acutely injure the cell membrane by influencing the stability of the intracellular metabolic environment. REC in present study reflects alteration in the permeability of plant cells and the degree of cell membrane damage under salinity. The greater damage the plant will suffer, the higher REC is (Dinneny, 2015). Similarly, as an end product of reactive oxygen and membrane lipid, the content of MDA also reflects the degree of cell damage (Wha et al., 2020). Our data show that the REC of *X. sorbifolium* increased sharply upon increasing salt concentration. It demonstrates that salt stress destroys the cell membrane of the plant leaves, leading to the outflow of osmotic substances, and thus the increase of the REC and MDA. The damage from slight concentrations of NaCl can be relieved by the salt tolerance of the plants itself, while the AMF symbiotic benefit played a more important role under high salt stress (above 240 mM). Although performance of MDA in AM plants was not ideal in response to 160 mM, we believe that the alleviation of AMF began to produce a marked effect above 160 mM, as the trend of SOD also proved this point of view.

0Plants can adjust the osmotic balance of cells by synthesizing osmotic regulating substances to increase their own salinity tolerance, thereby alleviating the damage caused by salt stress (Sperdouli and Moustakas, 2012). In NM plants, the regulation of proline almost depended on plant resistance itself under 240 mM NaCl. Proline of NM plants reached the maximum upon 240 mM NaCl and decreased at 320 mM NaCl. Therefore, salt-resistant ability is the strongest upon 240 mM NaCl and then turns to downward trend under the highest concentration. In contrast, the proline of AM plants peaked under 320 mM NaCl, and the value is higher than corresponding NM plants, which showed that AM plants possess stronger resistance. Plants can resist salt stress by accumulating soluble protein and proline, and AMF can effectively alleviate the damage of osmotic metabolism system caused by high concentrations of NaCl (Dudhane et al., 2011; Todea et al., 2020). Studies have shown that AMF can maintain a low intracellular osmotic potential and normal cellular metabolism by promoting the accumulation of proline in mycorrhizal plants under salt stress (Feng et al., 2002). However, when treated with 80, 160, and 240 mM NaCl, proline of AM plants is lower than NM plants. It may be explained by delayed effect of AMF (activated only at higher concentrations), and it is close to the result of MDA.

By maintaining the rapid and effective operation of ASA-GSH cycle, plants can effectively reduce the reactive oxygen species (ROS) under stress (Miller et al., 2010). In this study, the GSH and ASA of NM plants increased significantly at the concentration of 160 and 240 mM NaCl in comparison with control plants in NM group, but they significantly decreased at 320 mM NaCl. It reflects the role of antioxidants in alleviating salt damage, but the function is limited. For AM plants exposed to NaCl concentrations above 160 mM, the GSH and ASA content continued to increase. It is likely that mycorrhizal fungi on average accelerate the process of ASA-GSH cycle (Andres and Andrea, 2002). Similar results showed that AMF can enhance ASA-GSH cycle in many plants (Degola et al., 2015; Yang H. et al., 2015). This study suggests that *X. sorbifolium* may protect the plant mainly by increasing GSH and ASA content to eliminate ROS in response to salt stress.

Salinity-induced reduction in plant growth is often related to a downregulation in their photosynthetic capacity (Ashraf et al., 2009). In this study, in the AM and NM plants, Pn showed a trend of first increasing and then decreasing, resulting from increasing NaCl concentrations. In addition, Gs, Ci, and E declined significantly compared with corresponding control plants. It can be deduced that high concentrations of salinity could damage the photosynthetic mechanism and decrease the electron transfer rate (Logan et al., 1999), which can be concluded as a non-stomatal factor (Fu Y. et al., 2008). Under 320 mM NaCl, the seedlings could no longer carry out normal photosynthesis. Moreover, AM plants have higher Pn, Gs, and Ci across all treatments as compared with NM plants, which can be explained from the perspective of CO_2_ diffusion and assimilation process (Chen S. et al., 2017). After inoculation, the openness of stomatal aperture is greater and CO_2_ diffusion is quicker, which contribute to the normal photosynthesis of plants.

The light response curve describes the relationship between the quantum flux density of light and the Pn of plants. The lower Pn of salt-stress seedlings is probably connected with low water potential around the rhizosphere, which may induce the closure of stomata (Terzi et al., 2014). Under high salt stress (240 and 320 Mm NaCl), the Pn of AM plants initially increased upon increasing PAR, but when the PAR exceeded 1,200 μmol·m^−2^ s^−1^, the Pn of AM plants gradually decreased, possibly because AMF alleviated the damage caused by salt stress to plants, and resulted in enhanced photorespiration or photoinhibition caused by high light intensity (Borkowska, 2006). At 160 mM of salt stress, NM plants showed a trend of increasing light intensity at about 1,500 μmol·m^−2^ s^−1^. The possible reason for that is that the photosynthetic mechanism of plants is destroyed when the salt damage is severe, and the synergistic effect of AMF and strong light may promote the transfer of photoelectrons and improve the efficiency of light energy utilization (Porcel et al., 2015). AQY could describe the photosynthetic efficiency under low light intensity (Lambers and Oliveira, 2019). Reduction of AQY and Pnmax in our study under high salt stress may be due to the decline of Calvin cycle efficiency, especially the utilization of ATP and NADPH (Yang et al., 2010). Moreover, under high concentrations, LSP shows a downward trend and LCP shows an upward trend, and the phenomenon may be attributed to the more limiting processes such as electron transport and the metabolism of triose phosphates (Yang et al., 2010). These observations indicated that high salt concentration had a strong inhibitory effect on the photosynthetic ability of *X. sorbifolium*. Besides, under salt stress, excessive Na^+^ in the thylakoid membrane inhibits the activity of PSI and PSII (Allakhverdiev et al., 2000), which leads to the change of photosynthetic mechanism and causes the destroy of structure and function of chloroplast. In brief, AMF can maintain the ion homeostasis of roots to a certain extent, prevent excessive Na^+^ ions from entering, and reduce the degree of damage to the photosynthetic function of *X. sorbifolium* under salt stress.

Antioxidant enzymes can reduce the harmful effect of oxidative stress by scavenging excessive reactive oxygen species (ROS; Hashem et al., 2015). Salinity-induced H_2_O_2_ production causes disorder in intercellular homoeostasis and promote the electrolyte leakage resulting from greater membrane lipid peroxidation (Alqarawi et al., 2014). It can be observed that in NM plants, SOD decreased above 240 mM NaCl, while POD and CAT declined similarly under 320 mM NaCl (Figure 8). Antioxidant enzymes in AM plants showed an upward trend below 240 mM NaCl. The phenomenon showed that the tolerance of oxidative stress is limited in *X. sorbifolium,* likely because there were not enough antioxidant enzymes activity to scavenge excessive ROS (Yang et al., 2014). More production of SOD can accelerate the reaction of superoxide radicals into H_2_O and H_2_O_2_, which are then transformed into H_2_O and O_2_ molecules catalyzed by CAT (Mittler, 2002). In general, the mycorrhizal had the most impact on SOD and CAT among these antioxidant enzymes. Moreover, current data showed that there is higher activity of antioxidant enzymes in mycorrhizal treatments compared with NM plants under 320 mM NaCl, and it can be corroborated with previous findings (Tang et al., 2009; Latef and He, 2011; Wu et al., 2016; Huang et al., 2017; Hashem et al., 2018). It can be speculated that AMF colonization is beneficial to the synthesis of immune-related enzymes and enhances the antioxidant enzyme defense system (Yang Y. et al., 2015). Furthermore, given that the increase of SOD and CAT in AM plants in comparison with NM plants has reached their maximum under 320 mM, we can conclude that among various antioxidant enzymes, SOD and CAT played a crucial role in the elimination of ROS (Foyer et al., 2010), especially under high concentrations. In conclusion, AMF may resist salinity to protect plants by alleviating the NaCl induced oxidative stress.

In general, our results support the view that the beneficial effects of AM symbiosis can contribute to better growth performance of *X. sorbifolium* upon exposure to salinity by accumulating osmotic regulating substances, enhancing SOD and POD enzyme activity and photosynthetic capacity, as well as reducing the root allocation of Na^+^. The increasement in photosynthesis by AM symbiosis was correlated with a lower reduction in Gs, a higher and Ci in comparison with NM plants. Notably, the improvement of CAT enzyme activity and accumulation of osmoregulation substances and antioxidants play a more important role in AM plants against high level salinity stress.

## 5. Conclusion

As noted above, the symbiosis between a *X. sorbifolium* tree and AMF species (*Funneliformis mosseae*) were well established under salt stress conditions. As a salt resistant species, *X. sorbifolium* can adapt to the damage from salinity by accumulating osmotic adjustment substances, increasing SOD and CAT activity. However, under high concentrations of NaCl (240 and 320 mM), the resistant ability of the plant significantly decreased, as evidenced by the significant decrease of Pn and biomass. It indicates that the regulatory capacity of *X. sorbifolium* against salinity is limited, and it played an important role only up to certain amount of NaCl. After inoculation of AMF, Gs of plant leaves increased and Ci declined compared with NM plants, while Pn and Pnmax were downregulated as well. Under high salt stress, GSH, ASA, proline, and soluble protein of AM plants are higher in comparison with NM plants, revealing that mycorrhizal symbiotic benefits were more crucial against severe salinity toxicity.

## Data availability statement

The original contributions presented in the study are included in the article/supplementary material, further inquiries can be directed to the corresponding author.

## Author contributions

JZ and YY conceived the ideas, designed the methodology, and initiated preparation of the manuscript. ZZ and PH collected data, analyzed the data, and revised the manuscript. JZ led the writing of the manuscript. All authors contributed to the article and approved the submitted version.

## Funding

This study was supported by the Science and Technology Project of Henan Province (Project No. 232102110179), the Key Projects of Provincial Universities in Henan Province (Project No. 19A220002), and the Doctoral Research Start-Up Fund project funding (2018HNUAHEDF018 and 2018HNUAHEDF019).

## Conflict of interest

The authors declare that the research was conducted in the absence of any commercial or financial relationships that could be construed as a potential conflict of interest.

## Publisher’s note

All claims expressed in this article are solely those of the authors and do not necessarily represent those of their affiliated organizations, or those of the publisher, the editors and the reviewers. Any product that may be evaluated in this article, or claim that may be made by its manufacturer, is not guaranteed or endorsed by the publisher.
